# Pepper-YOLO: an lightweight model for green pepper detection and picking point localization in complex environments

**DOI:** 10.3389/fpls.2024.1508258

**Published:** 2024-12-31

**Authors:** Yikun Huang, Yulin Zhong, Deci Zhong, Changcai Yang, Lifang Wei, Zhoupei Zou, Riqing Chen

**Affiliations:** ^1^ School of Future Technology, Fujian Agriculture and Forestry University, Fuzhou, China; ^2^ Concord University College, Fujian Normal University, Fuzhou, China; ^3^ Center for Agroforestry Mega Data Science, Fujian Agriculture and Forestry University, Fuzhou, China

**Keywords:** green pepper detection, Pepper-YOLO, picking point localization, lightweight model, picking robot

## Abstract

In the cultivation of green chili peppers, the similarity between the fruit and background color, along with severe occlusion between fruits and leaves, significantly reduces the efficiency of harvesting robots. While increasing model depth can enhance detection accuracy, complex models are often difficult to deploy on low-cost agricultural devices. This paper presents an improved lightweight Pepper-YOLO model based on YOLOv8n-Pose, designed for simultaneous detection of green chili peppers and picking points. The proposed model introduces a reversible dual pyramid structure with cross-layer connections to enhance high-and low-level feature extraction while preventing feature loss, ensuring seamless information transfer between layers. Additionally, RepNCSPELAN4 is utilized for feature fusion, improving multi-scale feature representation. Finally, the C2fCIB module replaces the CIB module to further optimize the detection and localization of large-scale pepper features. Experimental results indicate that Pepper-YOLO achieves an object detection accuracy of 82.2% and a harvesting point localization accuracy of 88.1% in complex scenes, with a Euclidean distance error of less than 12.58 pixels. Additionally, the model reduces the number of parameters by 38.3% and lowers complexity by 28.9%, resulting in a final model size of 4.3MB. Compared to state-of-the-art methods, our approach demonstrates better parameter efficiency. In summary, Pepper-YOLO exhibits high precision and real-time performance in complex environments, with a lightweight design that makes it well-suited for deployment on low-cost devices.

## 1 Introduction

In modern agriculture, automated harvesting has become an important means to improve production efficiency and reduce labor costs. Peppers, as a significant economic crop, hold an important position in the cultivation and harvesting in many regions. However, current pepper harvesting robots face numerous challenges in complex agricultural environments, such as color similarity, dynamic lighting, and severe occlusion, which result in subpar performance of existing detection technologies in practical applications. In previous work, many researchers have attempted to enhance detection accuracy by increasing model complexity; however, complex models require higher equipment costs. Therefore, improving the recognition accuracy of peppers and harvesting points while reducing model complexity is crucial for enhancing the level of agricultural automation (Hua et al., 2023).

In complex environments, peppers are often occluded by leaves, branches, or even other peppers, which significantly impairs both object detection and the localization of picking points. Furthermore, compared to fruits with stable contours such as bell peppers, apples, and oranges, green peppers exhibit irregular surfaces and complex morphologies, including straight, curved, and twisted shapes. These factors present considerable challenges for the recognition tasks of harvesting robots. Since the early days of object detection, hand-crafted feature extraction methods and classifiers, such as SIFT and HOG, have been combined with Support Vector Machines (SVM) for object classification (Bellavia and Colombo, 2020; Zhou and Yu, 2021). Arad developed a highly regarded sweet pepper-picking robot that uses the color difference between yellow peppers and the background for segmentation and employs the Hough transform for key point localization (Arad et al., 2020). Ji et al. proposed a local contrast enhancement algorithm to enhance green pepper images, followed by boundary pixel information for edge detection, achieving an accuracy of 83.6% (Ji et al., 2020). Bai et al. constructed a machine learning model to recognize tomatoes using Hough Circle Detection, leveraging shape, texture, and color features (Bai et al., 2023). Zhu et al. achieved grape instance segmentation using components of the HIS color space and the Otsu algorithm (Zhu et al., 2022). While these methods perform adequately in simple backgrounds or fixed scenes, they often struggle with complex backgrounds, multiple occlusions, varying scales, and object angles. Additionally, traditional methods require significant human intervention and feature engineering, leading to poor scalability and limited adaptability in complex agricultural environments.

In recent years, with the advent of convolutional neural networks (CNN) and advancements in deep learning algorithms, object detection technology has been widely applied in agriculture. Currently, object detection is categorized into two-stage and one-stage detectors. Two-stage detectors typically employ a complex network structure to first generate region proposals, followed by feature extraction and detection for each region. Representative models include R-CNN and Faster R-CNN (Wan and Goudos, 2020). For example, Wang et al. (Wang et al., 2022) used ResNet-50 as the backbone for Faster R-CNN, providing precise recognition for automated harvesting robots through two-stage detection. In contrast, one-stage detectors perform regression and classification tasks directly on the feature map of the image without an explicit region proposal step, enabling faster detection speeds compared to two-stage detectors (Diwan et al., 2023). Typical examples of single-stage detectors include the YOLO series and SSD.

One-stage detectors, with their end-to-end network architecture, excel in real-time object detection and have been widely applied in agriculture, such as in plant phenotyping (Jiang and Li, 2020), localization in fruit picking (Chen et al., 2024c), tomato leaf disease detection (Tang et al., 2023), and small apple detection in orchards (Sun et al., 2022). In picking point localization, many researchers have combined one-stage detectors with traditional image processing and geometric methods to achieve stem recognition and key point localization. For example, in strawberry picking point detection, Yu et al. measured and annotated the rotation angle of each strawberry during data labeling, and based on YOLOv3, proposed the R-YOLO model. This method estimates the strawberry’s posture by predicting the rotation angle of the bounding box and then uses statistical methods to predict the picking point location (Yu et al., 2020). Similarly, in strawberry picking point identification, Tafuro et al. constructed a strawberry point cloud and used point cloud segmentation to estimate the picking point location (Tafuro et al., 2022). Although this method addresses the picking point localization problem, it requires a substantial amount of computational resources. Qi et al. combined YOLOv5 with traditional image processing techniques to accurately identify the main stems and picking points in lychee images (Qi et al., 2022). Zhang et al. used an improved YOLOv5 to detect grape centroids and then applied geometric estimation to determine the picking point; however, this approach overlooks the orientation and posture of the grape stems (Zhang et al., 2023). Although combining deep learning with geometric methods can estimate picking point locations, these methods struggle to accurately determine the correct picking points in environments with complex postures and occlusions.

To improve the accuracy of fruit detection in complex environments, multimodal image feature fusion has also been applied to object detection tasks. For instance, the fusion of RGB images and data from infrared camera sensors after image matching and integration can help solve the challenges of object detection in complex scenes (Liu et al., 2023). Similarly, the researchers solved the localization problem of the fruit by fusing RGB-D data with RGB images, which can also alleviate the problem of overlapping occlusion in complex environments (Zhou et al., 2024). Several lines of evidence suggest that the fusion of multimodal data can provide richer feature information. However, multimodal data fusion not only requires more memory and computational power, but also must overcome feature conflicts between different data modalities (Liu et al., 2023; Chen et al., 2024d).

With the development of deep learning and pose estimation technologies, models for parallel computing of object detection and pose estimation have gained significant attention. Some researchers have applied the YOLO-Pose model in the field of agricultural harvesting. For example, Chen et al. utilized an improved YOLOv8-pose model to achieve object detection and keypoint localization for strawberries (Xia, 2024). Similarly, Huang et al. employed an improved YOLOv8n-Pose model for object detection and harvesting point identification of grape clusters (Chen et al., 2024b). Although they have achieved success in parallel computing for object detection and keypoint localization using the YOLO-Pose series, the pyramid structure in their backbone is prone to feature loss during multi-scale feature fusion (Wang et al., 2024), resulting in suboptimal detection accuracy for complex backgrounds or overlapping phenomena. In summary, despite the progress made in fruit object detection and picking point localization, several challenges remain. First, object detection and picking point localization in complex environments is a complicated process, and current automated picking systems lack detection networks that are both lightweight and efficient. Second, due to the diverse shapes of green peppers and various occlusion issues, existing algorithms struggle to achieve satisfactory accuracy in occlusion recognition. Lastly, many pyramid-based multi-layer network structures suffer from feature loss, resulting in low reliability when detecting multi-scale objects.

**Figure 1:**
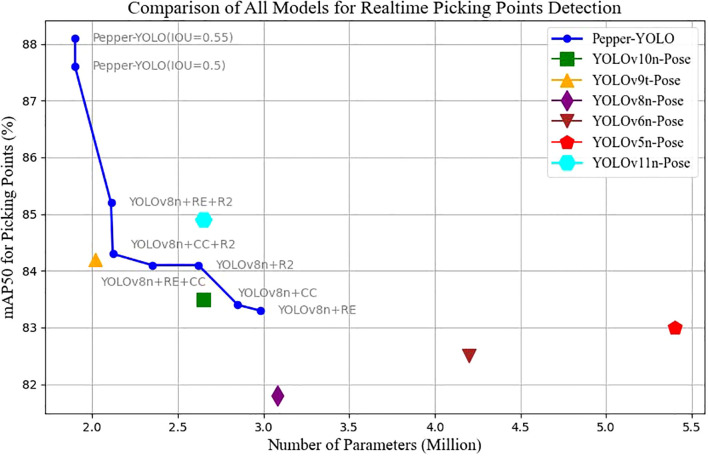
Pepper-YOLO is a method we proposed for simultaneous green pepper detection and picking point localization. Compared to state-of-the-art methods, Pepper-YOLO achieves the best performance while maintaining the smallest model size(only around 1.9M parameters).

Improving the efficiency of automated green pepper harvesting requires not only detecting the peppers and localizing picking points but also overcoming challenges such as occlusions and varying lighting conditions in complex environments. This paper proposes a lightweight and efficient Pepper-YOLO model for simultaneous detection of green pepper targets and picking point localization. Specifically, our goal is to enhance the accuracy of pepper object detection and picking point localization while reducing the model’s computational complexity to ensure efficient operation on resource-constrained hardware. In addition, this model integrates the detection of multiple keypoints of pepper postures, which can also serve for pepper posture recognition. We conducted experiments using a dataset collected from real-world scenarios, and the experimental results demonstrate that our proposed Pepper-YOLO achieved the best performance in all comparisons, as shown in **Figure 1**.

The contributions of this paper are manifold:

- Establish a green pepper dataset that includes object detection bounding boxes, picking points, and posture keypoints to assist with harvesting.
- The use of a reversible detection head with a double pyramid structure reduces information loss during network transmission and improves multi-scale object detection accuracy.
- Introduce a lightweight model, Pepper-YOLO, which combines pepper object detection with picking point localization, enabling simultaneous detection of green peppers and their keypoints, while demonstrating superior accuracy and fewer parameters compared to several state-of-the-art (SOTA) deep networks.

## 2 Materials and methods

### 2.1 Dataset acquisition

**Figure 2:**
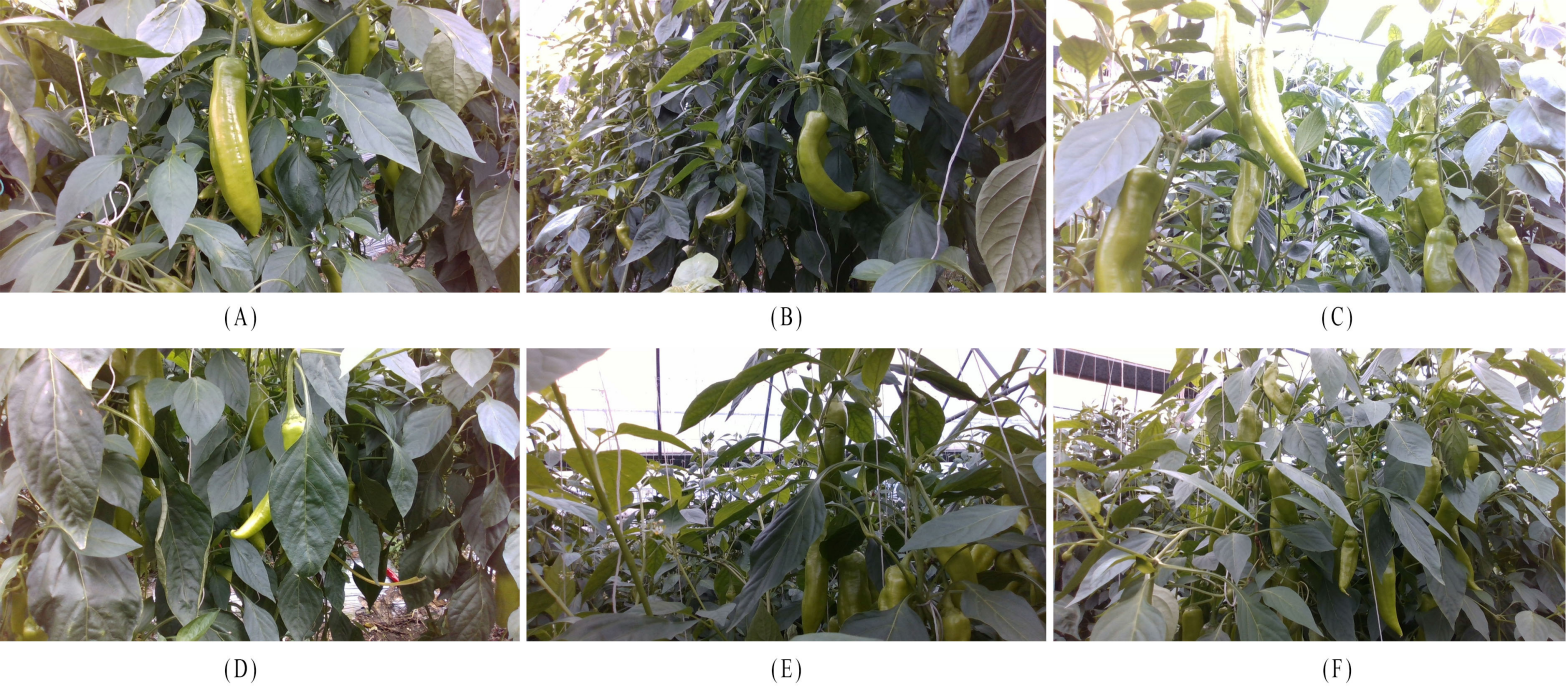
Peppers in complex scenes with different shapes, lighting conditions, and types of occlusion. **(A)** Straight pepper, **(B)** Curved pepper, **(C)** Backlit pepper, **(D)** Pepper occluded by leaves, **(E)** Pepper occluded by branches, **(F)** Peppers occluding each other.

To evaluate the performance of the model in complex environments, data was collected on May 2, 2024, at the green pepper plantation of Lvfeng Agricultural Technology Co., Ltd. in Fuqing City. During this period, the pepper plants were at their most foliated, with significant occlusion of the peppers, accurately representing complex real-world conditions. Data was captured using an Intel RealSense D435i camera, which consists of an infrared camera, dot projector, and RGB camera, capable of depth measurement within a range of 0.2 to 2 meters. During the capture process, the distance between the camera and the peppers was maintained at 50 to 70 cm. A total of 1,152 images, including both RGB and depth images, were collected, containing 2,381 individual peppers, with a resolution of 1280x720. The dataset covers both front-lit and backlit environments. As shown in **Figures 2A–F**, the peppers in the dataset exhibit various shapes, including straight, curved, long, and short peppers. These peppers experienced multiple types of occlusion, such as leaf occlusion, inter-pepper occlusion, branch occlusion, and cases where only part of the pepper was visible in the image.

### 2.2 Datasets annotation

**Figure 3:**
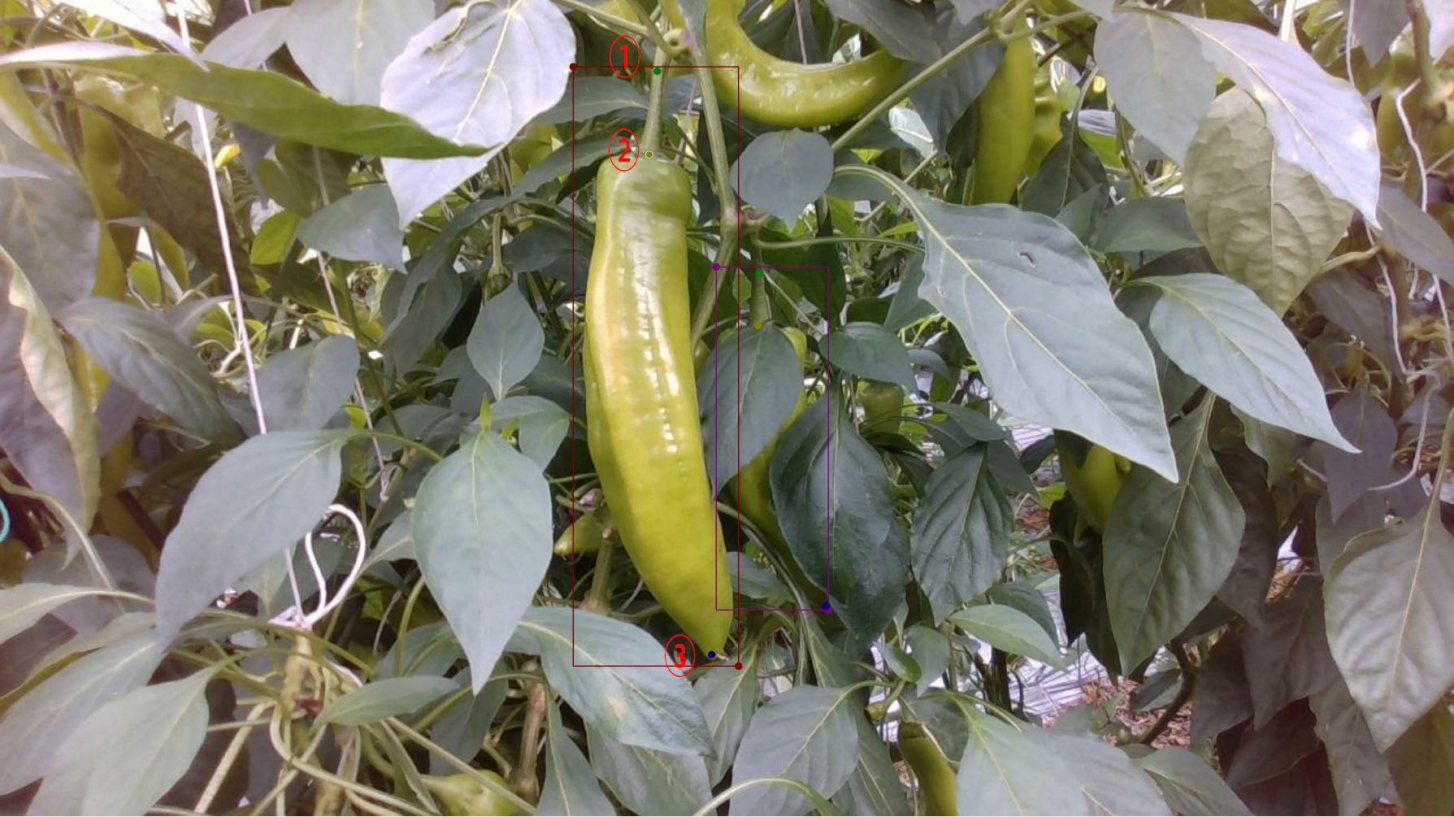
Green pepper target bounding box and the annotated locations of the three keypoints.

The images were annotated using Labelme, where rectangular boxes were used to mark the pepper regions, and keypoints were created at three locations: the stem, the top of the fruit, and tip of the pepper. The purpose of annotating three keypoints for each pepper is to correct the picking point location and make an initial assessment of the pepper’s shape. The annotation process follows these principles: (1) The stem keypoint, representing the picking point, is located 2cm to 5cm above the pepper and is labeled as “Pick,” as shown in **Figure 3** by number 1. (2) The localization point at the top of the fruit is placed in the center and is labeled as “Top”, as shown in **Figure 3** by number 2. (3) The tip of the pepper is labeled as the “Bottom” keypoint, as shown in **Figure 3** by number 3. (4) Each keypoint can be categorized into three visibility states: the “Visible” state, where the location point is clearly identifiable in the image; the “Occluded” state, indicating the location point is partially or completely obscured by leaves, stems, or other parts of the pepper; and the “Invisible” state, in which the location point is entirely outside the captured range of the image.

### 2.3 Dataset construction

The annotated dataset was divided into training, validation, and test sets in a ratio of 7:1.5:1.5. The validation set contains 86 images with a total of 328 peppers, and the test set contains 87 images with a total of 410 peppers. To ensure the model learns feature representations in complex environments and adapts to varying lighting conditions, offline data augmentation techniques were applied to randomly selected images from the training set. These augmentations included mirroring, translation, brightness adjustment, and scaling, expanding the training set to 1,261 images with a total of 4,797 peppers.

### 2.4 Overview of YOLOv8-Pose

YOLOv8-Pose is one of the latest variants in the YOLO series, specifically designed for object detection and pose estimation tasks (Varghese and M, 2024). YOLO models have gained significant attention in the field of object detection due to their fast and efficient performance. Building on this, YOLOv8-Pose further enhances pose estimation capabilities (Si et al., 2023). The model combines the efficiency of the YOLO architecture with the advantages of multi-task learning, allowing it to accurately detect objects and estimate keypoints while maintaining real-time performance. Compared to YOLOv5 (Jocher, 2020), YOLOv6 (Li et al., 2022), and YOLOv7 (Wang et al., 2023), YOLOv8 demonstrates superior performance across multiple datasets.

The backbone of YOLOv8 consists of a series of convolutional layers and improved CSPNet modules (such as the C2f module), responsible for gradually extracting multi-scale features. Through downsampling and feature fusion, it generates feature maps at different scales (e.g., P3, P4, P5), providing rich feature representations for subsequent object detection. In the neck network, the C2f module further integrates and transmits these features (Chen et al., 2024a), utilizing structures like the feature pyramid network (FPN) to effectively process features from different scales, thereby improving the detection of objects of varying sizes. Before generating bounding boxes and keypoint coordinates, YOLOv8 continues to use the C2f module for multi-layer feature fusion, ensuring the accuracy of the final detection results. This network architecture is highly effective for fruit detection and picking point localization tasks in agricultural and industrial environments (Yang et al., 2023; Diao et al., 2023).

### 2.5 Reconfiguring the backbone network

**Figure 4:**
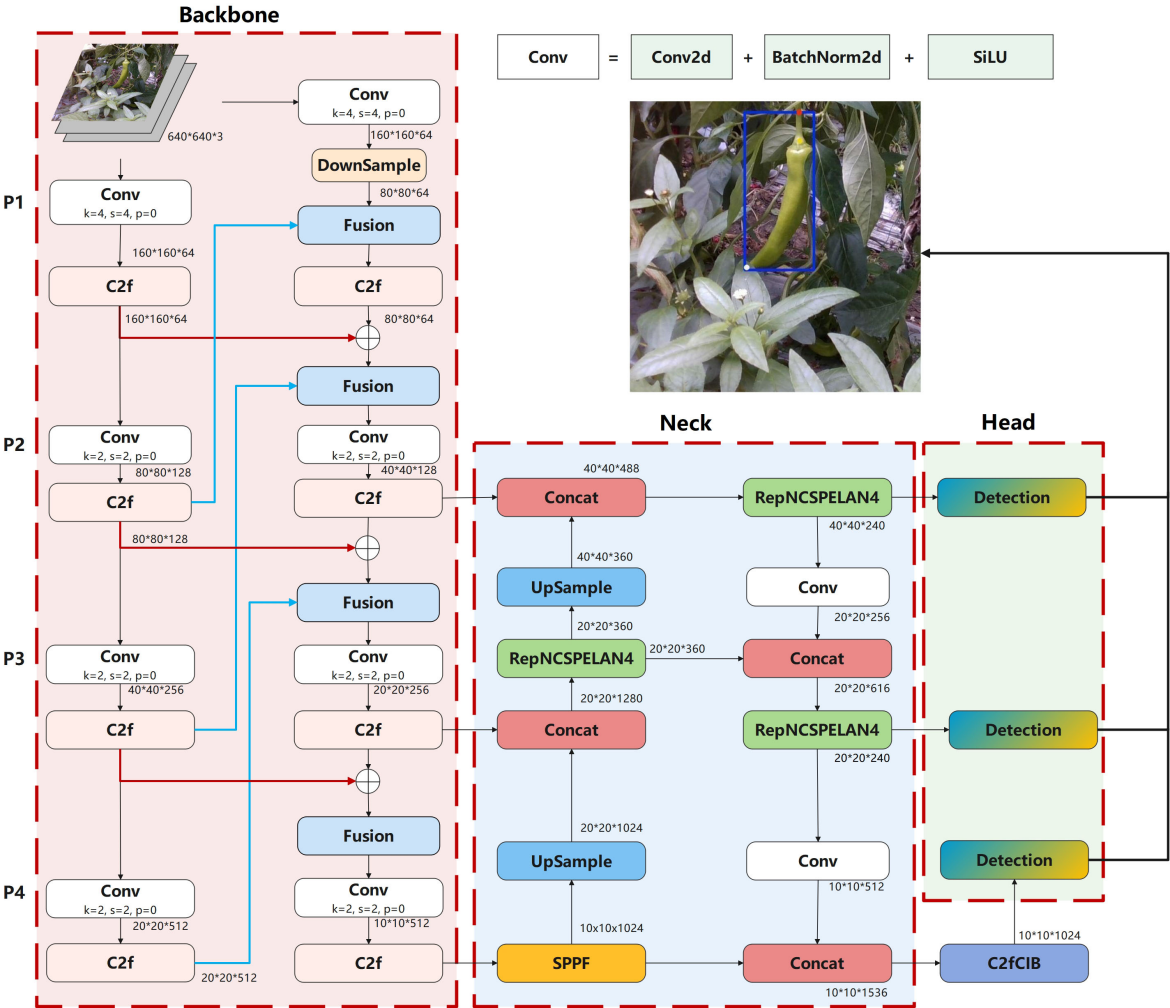
Network structure of Pepper-YOLO.


YOLO typically uses a top-down and bottom-up pyramid structure for feature fusion, which has significant advantages in feature extraction (Quan et al., 2023). However, this structure tends to lose low-level feature information when extracting high-level features, especially in complex scenes with varying background and heavy occlusion, where the input feature resolutions differ, leading to inconsistent feature fusion contributions and reduced model performance (Kim et al., 2021). To address this issue, this paper proposes a backbone network named Rev2, which is composed of two bottom-up pyramid structures. Each of these pyramid structures takes the original image as input. Each pyramid structure consists of four levels, labeled as P1, P2, P3, and P4 in **Figure 4** backbone. The resolution of the P1 feature layer is 160×160, and each level uses different convolutional kernel sizes and strides for feature extraction and down-sampling operations, while the resolution of the P4 feature layer is 20×20. In addition, the high-level and low-level features from the first pyramid structure are fused with the low-level and high-level features from the second pyramid structure, respectively. The fusion paths are shown by the red and blue arrows in **Figure 4** backbone.

(1) $$F_{2}^{l+k}=F_{2}^{l+k}+T\left(F_{1}^{l}\right)$$

(2) $$F_{2}^{l}=F_{2}^{l}+T^{\prime }\left(F_{1}^{l+k}\right)$$

The mutual fusion of high-level and low-level features between the two pyramids during feature fusion is illustrated by Equations 1, 2 $F_{1}^{l+k}$ and $F_{2}^{l+k}$ . represent the high-level features of the two pyramids, while $F_{1}^{l}$ and $F_{2}^{l}$ represent the low-level features of the two pyramids, and *T* represents the feature transformation function used for spatial alignment.

Inspired by the concept of RevCol (Cai Y. et al., 2023), we apply reversible connections within the two pyramid structures, ensuring that features are not lost during both forward propagation and reverse restoration in the backbone network, as shown in Equations 3, 4. In this context, $f_{t}$ represents the nonlinear transformation operation. In Equation 3, the current feature map $F_{t}$ is generated by utilizing the previous layer’s feature map $F_{t-1}$ , along with $F_{t-m+1}$ from the earlier *m* + 1 layers and the weighted $F_{t-m}$ . This approach ensures that each layer in the deep network fully leverages the contextual information from different levels, thereby enhancing the feature representation capacity and overall network performance.


(3) $$F_{t}=f_{t}\left(F_{t-1},F_{t-m+1}\right)+\text{γ}F_{t-m}$$

(4) $$F_{t-m}={\text{γ}}^{-1}\left[F_{t}-f_{t}\left(F_{t-1},F_{t-m+1}\right)\right]$$

In Equation 4, the reversible operation ${\text{γ}}^{-1}$ is employed to recover the previous feature $F_{t-m}$ from the current layer’s feature $F_{t}$ . This design guarantees lossless feature transmission within the deep network. Specifically, during gradient calculation and parameter updates, it eliminates the need to store all intermediate activations, thus reducing memory consumption.

In addition, within the dual-pyramid structure, we use convolutional kernels of varying sizes and strides for feature extraction and downsampling. During feature transmission, we employ the lightweight C2f module to fuse and decouple partial features, enabling efficient multi-scale feature extraction while avoiding information redundancy and feature loss. Ultimately, through the fusion of features at different scales, we ensure that information is transmitted losslessly both forward and backward in the network, significantly improving feature extraction efficiency and detection accuracy.

### 2.6 Enhanced multi-scale feature fusion with RepNCSPELAN4 for pepper detection

In the green pepper cultivation environment, the variety of pepper shapes and occlusion issues result in peppers appearing at various sizes in images, which is one of the main challenges for pepper recognition and keypoint localization. Effectively fusing feature maps from different layers of the backbone and accurately identifying features at multiple scales become particularly important. In YOLOv8n, the neck uses the C2f module for feature fusion, but C2f’s fusion method is relatively straightforward, leading to potential information loss in deeper networks. This limitation is more pronounced in tasks requiring fine-grained information or very deep networks. Therefore, we replace C2f with RepNCSPELAN4 (Wang et al., 2024) to enhance the fusion of multi-scale features.

**Figure 5:**
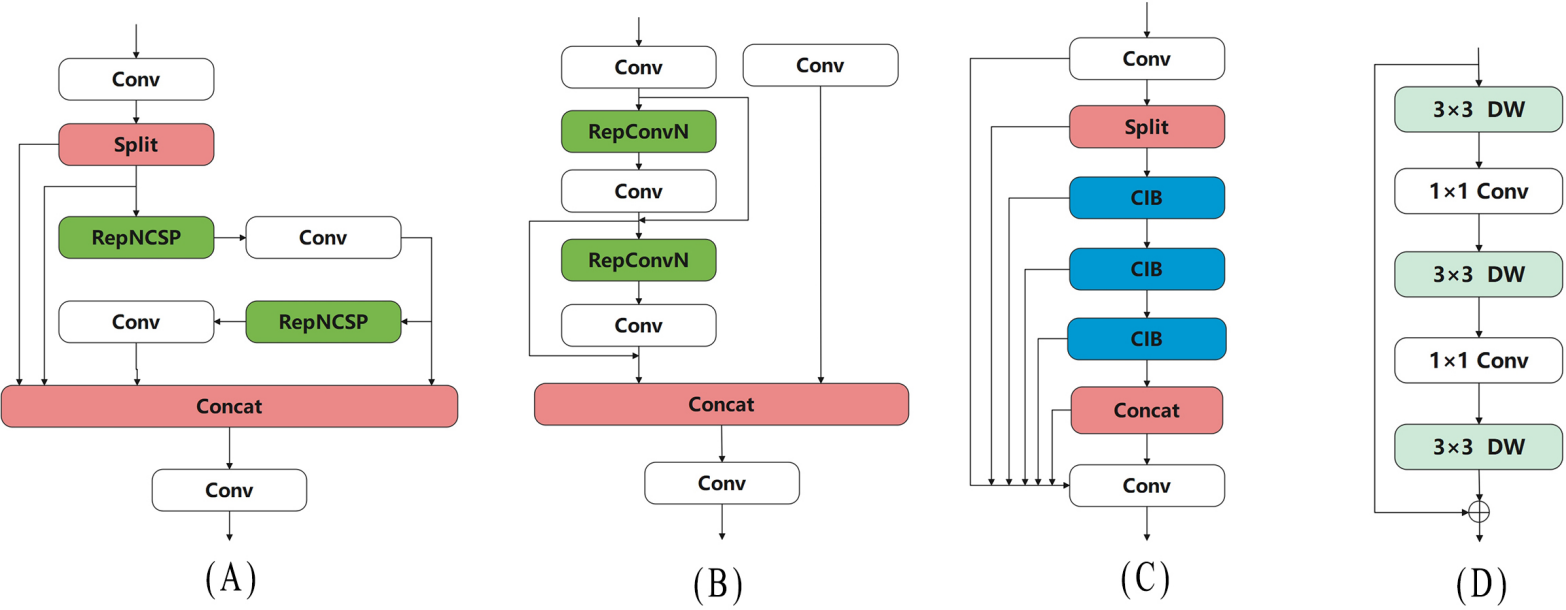
Network structure of Pepper-YOLO and its constituent modules. **(A)** RepNCSPELAN4, **(B)** RepNCSP, **(C)** C2fCIB, **(D)** CIB.

As shown in **Figure 5A**, RepNCSPELAN4 introduces multiple shortcut paths, allowing it to effectively retain information from different levels. This ensures that shallow feature information is not lost in deeper layers, thereby improving gradient flow stability. Additionally, RepNCSPELAN4 uses two RefConv (Cai Z. et al., 2023) layers in its RepNCSP structure to implement re-parameterization, and based on the CSPNet idea (Wang et al., 2020), it adopts an efficient layer aggregation strategy, which significantly reduces the model’s parameter count, as shown in **Figure 5B**. Through this feature aggregation strategy, RepNCSPELAN4 enhances the fusion of features across different scales, improving the detection capability for objects of varying sizes. Moreover, compared to other complex feature fusion modules, RepNCSPELAN4 reduces both the parameter count and computational complexity of the model, making it more efficient for deployment on resource-constrained devices, such as agricultural robots.

### 2.7 Enhancing large-scale feature recognition with C2fCIB

Green chili peppers have an elongated shape and occupy a large portion of the camera’s field of view. Overcoming occlusion and effectively recognizing large-scale features before the final inference is particularly important. To address this, we replaced the original C2f module with the C2fCIB structure in the penultimate layer of the network. C2fCIB builds on C2f by incorporating the Conditional Identity Block (CIB) to replace the bottleneck, as shown in **Figure 5C**. The CIB first applies Depthwise Convolution (Chollet, 2017), performing convolution on each input channel individually, followed by Pointwise Convolution (Hua et al., 2018) to restore the channel count. This interaction mechanism enhances feature information exchange without increasing computational costs, thereby improving feature extraction capabilities. The network structure is illustrated in **Figure 5D**. This multi-scale feature extraction method enables the model to handle objects of varying sizes more effectively. In complex scenarios, such as when object size varies significantly, C2fCIB outperforms C2f, particularly in processing targets with large-scale differences.

The main function of the C2fCIB module in Pepper-YOLO is to integrate features from different scales and layers, enhancing the network’s detection capabilities by increasing the number of channels and promoting feature interaction. Positioned in the penultimate layer, it plays a crucial role in improving the model’s ability to represent large-scale features, providing robust feature support for the final detection and localization tasks.

## 3 Experimental

### 3.1 Experimental details

To ensure the fairness of the experiments, all tests were conducted on the same hardware configuration. The experimental setup includes a GeForce RTX 4090D GPU, an AMD EPYC 9754 processor, and 60GB of RAM. The operating system used is Ubuntu 22.04, with PyTorch 2.0.0 as the development framework, CUDA 11.8, and Python 3.9 as the programming language. In this experiment, the input image size was standardized to 640×640. The hyperparameters for the experiment are as follows: the initial learning rate and the final learning rate were both set to 0.01, with a batch size of 8 images, and the momentum parameter was set to 0.937. Stochastic gradient descent (SGD) was selected as the optimizer, and the mosaic data augmentation was disabled during training. After 300 epochs of training, the best-performing weight file was extracted for model evaluation.

### 3.2 Evaluation metrics

To more accurately reflect the model’s potential in complex agricultural environments, we designed two core experimental stages: the first focuses on the precise detection of green peppers, and the second on the accurate localization of picking points. To quantify the model’s complexity, we used the total number of parameters (Params) and the number of giga floating-point operations per second (GFLOPs) as evaluation metrics, providing an in-depth analysis of the model’s computational burden and resource requirements. For measuring the model’s operational efficiency, we used frames per second (FPS) to assess the model’s ability to process video or image sequences in real-time. In evaluating the performance of the pepper detection task, precision (P), recall (R), and mean average precision (mAP) were utilized, with the definitions of these metrics provided in Equations 5–7.


(5) $$\text{Precision}=\frac{TP}{TP+FP}$$

(6) $$\text{Recall}=\frac{TP}{TP+FN}$$

(7) $$\text{mAP}=\overset{1}{\underset{0}{\int }}P\left(r\right)\;dr$$

TP (True Positives) refers to the number of instances where the model correctly detects and classifies the target object. FP (False Positives) represents the number of instances where the model incorrectly identifies an object, leading to false alarms. FN (False Negatives) indicates the actual number of target objects that the model fails to detect. mAP50 is a metric used to evaluate the model’s detection accuracy, representing the average precision across all categories when the Intersection over Union (IoU) threshold is 0.5. It takes into account both Precision and Recall. mAP50-95 is the mean of the average precision calculated over various IoU thresholds (ranging from 0.5 to 0.95 in steps of 0.05).


(8) $$X_{\text{pixel error}}=\left|x_{\text{pred}}-x_{\text{gt}}\right|\times \text{W}$$

(9) $$Y_{\text{pixel error}}=\left|y_{\text{pred}}-y_{\text{gt}}\right|\times \text{H}$$

(10) $$D_{i}=\sqrt{{\left(X_{\text{pred},i}-X_{\text{gt},i}\right)}^{2}+{\left(Y_{\text{pred},i}-Y_{\text{gt},i}\right)}^{2}}$$

(11) $$\sigma_{D}=\sqrt{\frac{1}{n}\overset{n}{\underset{i=1}{\sum }}{\left(D_{i}-\frac{1}{n}\overset{n}{\underset{j=1}{\sum }}D_{j}\right)}^{2}}$$

When evaluating the performance of the keypoint detection model, the commonly used error evaluation metrics are pixel error and Euclidean distance between two keypoints. Pixel error quantifies the difference by calculating the displacement between the predicted keypoint and the ground truth keypoint along the x-axis and y-axis. Specifically, $x_{pred}$ and $y_{pred}$ represent the normalized predicted coordinates, while *W* and *H* represent the width and height of the image, respectively. The pixel errors along the x-axis and y-axis are defined by Equations 8, 9. On this basis, the Euclidean pixel distance between the two keypoints is calculated using Equation 10. These metrics not only quantitatively reflect the discrepancy between the predicted and actual keypoint positions but also provide concrete guidance for model improvements. In the experiments, the average pixel error and average Euclidean distance across all instances were calculated to comprehensively evaluate the model’s overall performance under different test conditions. To assess the deviation of the Euclidean distances of individual points from the average Euclidean distance, Equation 11 is used to compute the standard deviation of the total Euclidean distance.

### 3.3 Ablation experiment

To evaluate the impact of different modules on the performance of the Pepper-YOLO model, we designed a series of ablation experiments. In these experiments, we sequentially removed or added the RepNCSPELAN4 (RE), C2FCIB (CC), and Rev2 (R2) modules. Both dual-module and single-module ablation experiments were conducted to thoroughly examine the contribution of each module to model complexity, computational efficiency, and detection performance. The experimental results are presented in **Table 1**.

**Table 1:** Ablation study on different modules.

RE	CC	R2	Params (M)	GFLOPs (G)	Size (M)	C50 (%)	P50 (%)	P95 (%)
×	×	×	3.08	8.3	6.4	78	81.8	76.5
✓	×	×	2.98	8.2	6.3	76.6	83.3	77.1
×	✓	×	2.85	8.2	6.0	79.1	83.4	76.8
×	×	✓	2.35	6.5	5.0	79.5	84.1	75.6
✓	✓	×	2.62	7.7	5.6	80.2	84.1	77.3
×	✓	✓	2.12	6.3	4.6	79	84.3	76.8
✓	×	✓	2.11	6.1	4.7	79.7	85.2	77.7
✓	✓	✓	1.9	5.9	4.3	81.9	87.6	80.4

RE, RepNCSPELAN4; CC, C2fCIB; R2, Rev2; C50, class mAP50; P50, Pose mAP50; P95, Pose mAP50-95.

The dual-module ablation experiment evaluates the effectiveness of individual modules without the support of the other two. In the experiment, when using only RepNCSPELAN4, C2FCIB, or Rev2, the Params and GFLOPs values decreased to varying extents, while both class mAP and picking point mAP improved. This demonstrates that the computational complexity was reduced in all cases. Notably, when using the Rev2 module, the Params of the model decreased by 23.7%, GFLOPs by 21.6%, and the model size reduced by 21.8%, indicating that Rev2, as the backbone, can extract more comprehensive feature information, significantly improving detection accuracy. From these results, we can conclude that all three modules not only reduce computational complexity but also enhance both class and picking point accuracy.

The single-module ablation experiment is designed to evaluate the impact of combining two modules on model performance. As shown in **Table 1**, when two modules are used together, they effectively reduce Params, GFLOPs, and model size, while also improving detection accuracy. Notably, when RepNCSPELAN4 is combined with the Rev2 module, the number of parameters is reduced by 31.5%. Finally, when all three modules are used simultaneously, the model achieves optimal performance, with Params reduced by 38.3%, GFLOPs by 28.9%, and model size by 32.8%. Additionally, class mAP@50 improved by 3.9%, while the picking point mAP@50 increased by 5.8% compared to the baseline, and mAP@50-95 improved by 3.9%. These experiments demonstrate that the Pepper-YOLO model effectively extracts feature information, reduces computational complexity, and enhances overall model performance.

### 3.4 Pepper detection comparative experiments

**Figure 6:**
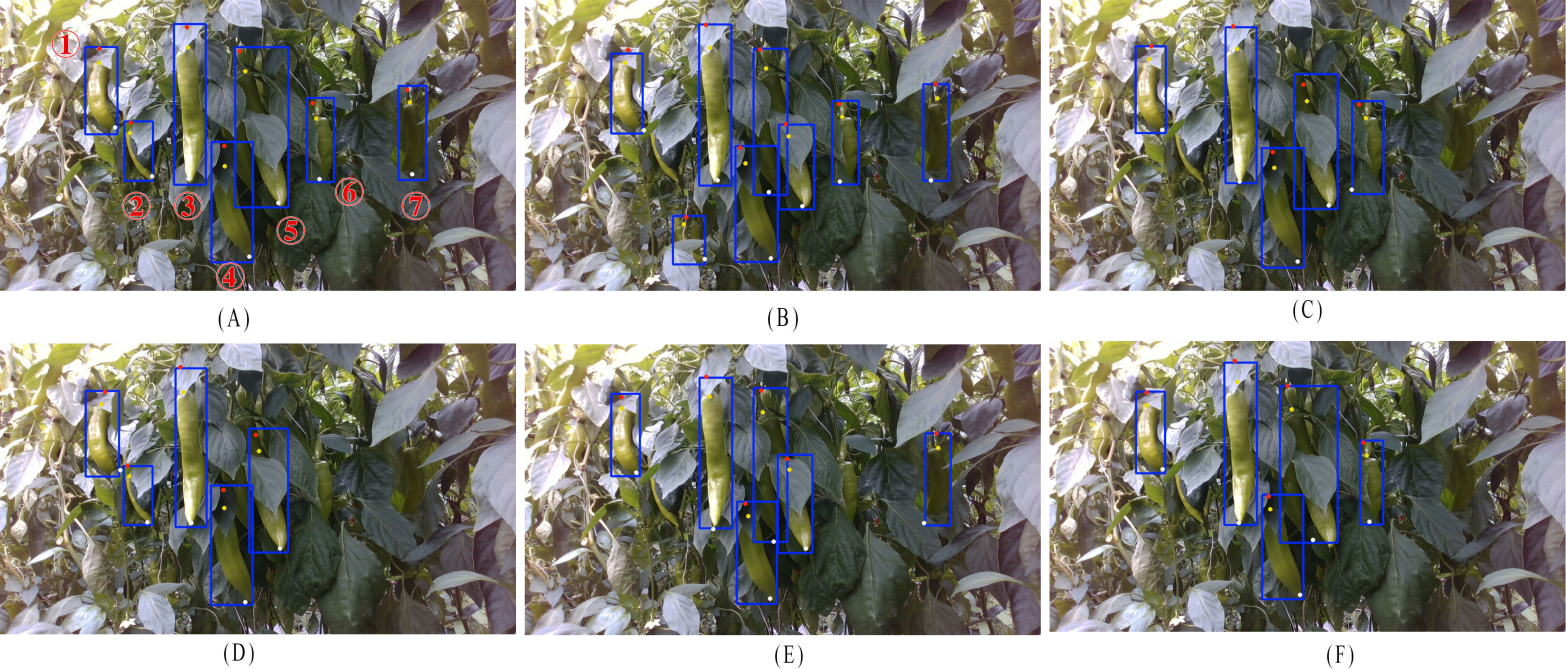
Detection results of different models for green peppers and the three keypoints. **(A)** Pepper-YOLO, **(B)** YOLOv10n-Pose, **(C)** YOLOv9t-Pose, **(D)** YOLOv8n-Pose, **(E)** YOLOv6n-Pose, **(F)** YOLOv5n-Pose.

To evaluate the comprehensive performance of Pepper-YOLO in green pepper detection under complex scenarios, we compared it with SOTA algorithms, with the test results shown in **Table 2**. Additionally, **Figure 6** visualizes the detection results of seven green pepper objects in a complex environment. In **Figure 5A**, the detected peppers are numbered. Due to issues such as occlusion, lighting, and color similarity, different models exhibit varied performance in these challenging scenarios. In **Figure 6A**, Pepper-YOLO successfully identifies all seven green peppers. Notably, Pepper-YOLO can detect pepper 1 despite its curved posture, and it accurately recognizes peppers 2, 5, and 6 under severe occlusion. Even under low light conditions, peppers 4 and 7 are correctly detected. Importantly, pepper 5, which is long, closely adjacent to other peppers, and heavily occluded by leaves, is still correctly recognized by Pepper-YOLO.

**Table 2:** Performance comparison of different algorithms.

Models	Params (M)	GFLOPs (G)	FPS	Size (M)	P	R	C50 (%)	P50 (%)	P95 (%)
YOLOv5n-Pose	5.4	7.3	263.2	5.5	80.1	68.5	79	83	77
YOLOv6n-Pose	4.2	11.8	277.8	8.8	83.9	65.9	78.7	82.5	76.3
YOLOv8n-Pose	3.08	8.3	294.1	6.4	78.0	53.1	78	81.8	76.5
Gold-YOLO	5.6	12.1	137.5	12.0	77.2	62.8	77.2	——	——
YOLOv9t-Pose	2.02	7.8	178.6	4.8	77.1	74.4	79.9	84.2	78.8
YOLOv10n-Pose	2.65	8	131.6	5.6	76.1	72.7	80.4	83.5	77.5
YOLOv11n-Pose	2.65	6.6	185.2	5.7	76.1	73.8	81.1	84.9	79.5
Pepper-YOLO	**1.9**	**5.9**	**500**	**4.3**	76.5	72.6	**81.9**	**87.6**	**80.4**
PY (iou=0.55)	1.9	5.9	500	4.3	75.5	73.2	**82.2**	**88.1**	**80.7**
PY (iou=0.60)	1.9	5.9	500	4.3	76.9	71.3	**82.0**	**87.8**	**80.6**

PY, Pepper YOLO; C50, class mAP50; P50, Pose mAP50; P95, Pose mAP50-95. Bold values indicate the highest (or best) performance values among the compared results.

In contrast, **Figure 6B** shows that YOLOv10n fails to correctly detect pepper 5, as the occlusion by leaves causes it to be mistakenly identified as two separate peppers. **Figures 6C, F** reveal that YOLOv9t and YOLOv5n-Pose are unable to detect peppers 2 and 7. While YOLOv9t does identify pepper 5, the occlusion prevents it from placing an accurate bounding box. In **Figure 6D**, YOLOv8n-Pose not only fails to correctly detect pepper 5 but also misses peppers 6 and 7 entirely. Lastly, in **Figure 6E**, YOLOv6n struggles to detect peppers 2 and 5, and fails to overcome the occlusion problem, which leads to incorrect identification of pepper 5. In summary, all the other algorithms exhibited both missed detections and false positives when detecting green peppers in complex environments, while Pepper-YOLO stands out as the most accurate in these challenging conditions.

In **Table 2**, Pepper-YOLO demonstrates exceptional performance and robustness in both object detection and keypoint localization tasks. Compared to other models, Pepper-YOLO has the smallest parameter count (1.9M) and lower computational cost (5.9 GFLOPs), enabling it to achieve excellent processing speed, reaching 500 FPS. Despite having fewer parameters, it maintains high accuracy in Pose mAP50 and Pose mAP50-95, achieving 87.6% and 80.4%, respectively, outperforming YOLOv11n-Pose (P50 = 84.9%), YOLOv10n-Pose (P50 = 83.5%), and YOLOv9t-Pose (P50 = 84.2%). Additionally, Pepper-YOLO exhibits strong robustness at different IoU thresholds (e.g., IoU = 0.55 and 0.60), with minimal variation in P50 and P95, demonstrating its stability under varying environmental conditions. Overall, Pepper-YOLO’s excellent performance in computational efficiency, detection accuracy, and robustness makes it an ideal real-time object detection model for green pepper.

In **Table 3**, the detection scheme using three keypoints (including the picking point, the top, and the bottom of the pepper) significantly improves the detection accuracy compared to the single picking point scheme. Both YOLOv10n-Pose and Pepper-YOLO show better performance with the three-point scheme, especially in terms of pose accuracy (P50 and P95). For Pepper-YOLO, with the three-point detection scheme, C50 increases from 80.7% to 81.9%, P50 rises from 78.0% to 87.6%, and P95 improves from 76.7% to 80.4%. This improvement indicates that adding more keypoints effectively enhances the accuracy and robustness of detection, particularly in pepper localization and pose estimation. In contrast, YOLOv10n-Pose shows only a slight improvement with the three-point scheme and still performs worse than Pepper-YOLO. Overall, the three-point detection scheme demonstrates a clear advantage in improving detection accuracy and robustness, and the superior performance of Pepper-YOLO in this configuration makes it a more reliable real-time object detection model.

**Table 3:** The detection comparison results of one point and three points were collected.

Models	P	R	C50 (%)	P50 (%)	P95 (%)
YOLOv10n-Pose (1 point)	81.0	69.0	79.3	77.5	76.2
YOLOv10n-Pose (3 points)	76.1	72.7	**80.4**	**83.5**	**77.5**
Pepper-YOLO (1 point)	73.5	75.4	80.7	78.0	76.7
Pepper-YOLO (3 points)	76.5	72.6	**81.9**	**87.6**	**80.4**

C50, class mAP50; P50, Pose mAP50; P95, Pose mAP50-95. Bold values indicate the highest (or best) performance values among the compared results.

### 3.5 Comparison experiment on the accuracy of pepper picking point recognition

**Figure 7:**
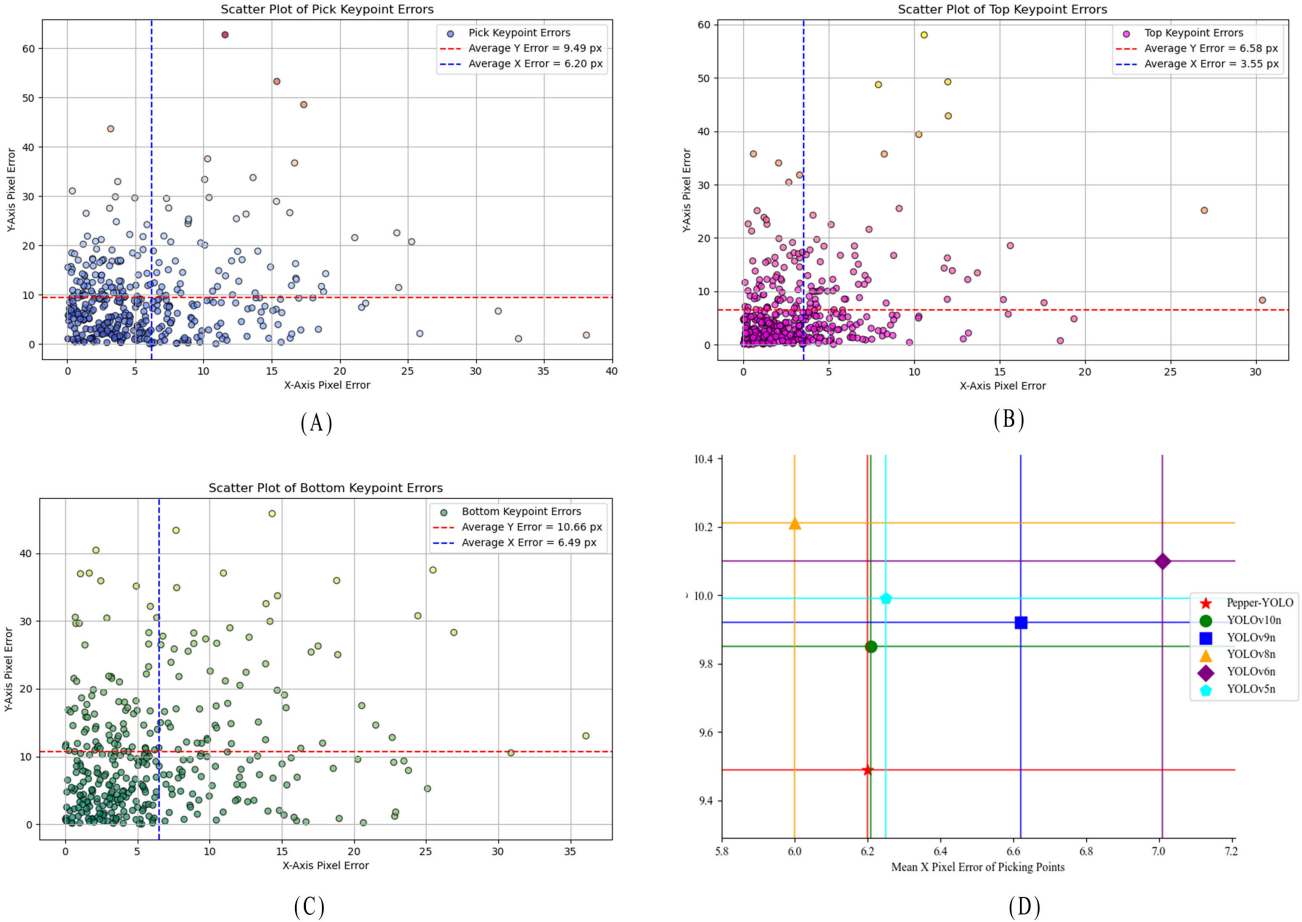
Distance between the three predicted points and the ground truth. **(A)** Pixel error statistical graph of the Pick keypoints for Pepper-YOLO, **(B)** Pixel error statistical graph of the Top keypoints for Pepper-YOLO, **(C)** Pixel error statistical graph of the Bottom keypoints for Pepper-YOLO, **(D)** Comparison of the average pixel error of the six models.

To evaluate the effectiveness and superiority of Pepper-YOLO in predicting the three key points of green peppers, we compared it with five other algorithms. As is well-known, different models may encounter missed detections and detection errors during the process. To ensure fairness, we selected 417 green pepper instances from a total of 738 peppers, all of which were correctly detected by every model, for comparative experiments. **Table 4** presents the pixel errors along the x-axis and y-axis for the “pick,” “top,” and “bottom” key points of the green pepper, along with the total average Euclidean distance error and the standard deviation of the Euclidean distance. Additionally, the scatter plot distribution of the pixel errors for the three predicted key points by Pepper-YOLO is shown in **Figure 7**. **Figures 7A–C** represent the scatter plots of the pixel errors for the picking point, calyx point, and tip point of the green pepper, respectively. In these figures, the x-axis denotes the horizontal error distance of the key points, while the y-axis represents the vertical error distance. The red dashed line indicates the average horizontal error, and the blue vertical line shows the average vertical error. Moreover, in **Figure 7D**, the different colored horizontal and vertical lines represent the average distance errors along the X and Y axes for different models in predicting the picking points of the green pepper.

**Table 4:** Results of comparative experiment of three points detection.

Models	Points	Average distance(pixel)			Standard deviation (pixel)
		X-axis	Y-axis	Euclidean distance	
Pepper-YOLO	Pick Top	6.2 **3.55**	**9.49** **6.58**	**12.58** **8.21**	**12.31** **11.22**
	Bottom	6.49	10.66	**13.65**	**13.40**
YOLOv10-Pose	Pick Top	6.21 3.37	9.85 7.48	12.74 8.9	14.30 13.67
	Bottom	6.92	**10.53**	13.99	17.2
YOLOv9t-Pose	Pick Top	6.62 4.19	9.92 7.64	13.12 13.12	14.30 14.48
	Bottom	7.44	11.66	15.1	19.82
YOLOv8n-Pose	Pick Top	**6.0** 3.58	10.21 7.27	12.94 8.96	14.66 14.21
	Bottom	6.61	10.81	13.99	16.38
YOLOv6n-Pose	Pick Top	7.01 4.05	10.1 8.29	13.63 10.11	14.84 13.76
	Bottom	7.44	11.93	15.33	18.74
YOLOv5n-Pose	Pick Top	6.25 3.58	9.99 7.94	12.93 9.46	13.85 14.21
	Bottom	6.47	11.49	14.41	17.47

Bold values indicate the highest (or best) performance values among the compared results.

From **Table 4**, it can be observed that although Pepper-YOLO’s average pixel distance on the X-axis for the “Pick” and “Bottom” key points is not as low as that of Yolov8n-Pose and YOLOv5n-Pose, its average pixel distance on the Y-axis outperforms all other models. Specifically, Pepper-YOLO achieves an average Euclidean pixel distance of 12.58 pixels, 8.21 pixels, and 13.65 pixels for the three key points, respectively. Furthermore, when calculating the standard deviation of the Euclidean pixel distance, Pepper-YOLO exhibits the lowest standard deviation for all three key points, indicating the smallest precision variance. **Figures 7A–C** show that most key points are concentrated below or to the left of the average distance error lines, indicating that the predicted points are generally below the average error threshold. In **Figure 7D**, a comparison of the average pixel errors in picking point localization across six algorithms is presented, demonstrating that Pepper-YOLO achieves the highest accuracy for the picking point. In practical harvesting tasks, the width of the robotic arm’s end-effector can range between 2-5 cm, allowing for some deviation in the picking point’s position. Based on practical experience, a pixel distance error of up to 30 pixels is acceptable for successful harvesting (Chen et al., 2024b). In summary, Pepper-YOLO’s prediction accuracy for the three key points meets the requirements for practical green pepper harvesting tasks.

## 4 Discussion

To address the challenges of chili pepper harvesting in complex agricultural environments, this study introduces the innovative Pepper-YOLO algorithm, which demonstrates exceptional performance in green chili pepper detection and picking point localization. Compared to existing state-of-the-art (SOTA) object detection algorithms, Pepper-YOLO achieves significant improvements in both accuracy and real-time performance, especially in handling complex scenarios such as peppers blending with the background, occlusions, lighting variations, and diverse fruit postures.

The core strength of Pepper-YOLO lies in its custom improvements based on the YOLOv8n-Pose model, particularly with the introduction of a reversible dual pyramid backbone network, which effectively integrates high-level and low-level features. This enhances the model’s ability to handle complex backgrounds and occlusions. Additionally, the integration of RepNCSPELAN4 feature fusion technology and the C2fCIB module further improves Pepper-YOLO’s ability to detect multi-scale targets, particularly in precisely locating pepper picking points. Compared to YOLOv8n-Pose, Pepper-YOLO’s localization accuracy increased by nearly 6 percentage points (as shown in **Table 2**). Even in heavily occluded scenarios, Pepper-YOLO can accurately detect and locate green chili pepper picking points (as illustrated in **Figure 6**), whereas traditional models often miss detections or generate false positives due to the complex background. Moreover, Pepper-YOLO uses three RepNCSPELAN4 structures, with output channels of 360, 240, and 240. In an experiment to capture higher-level semantic information, we attempted to adjust the third RepNCSPELAN4’s output channel to 360, but this led to a decline in overall accuracy. This suggests that an excess of features caused information redundancy or interference from irrelevant features, which affected feature selection and reduced accuracy.

**Figure 8:**
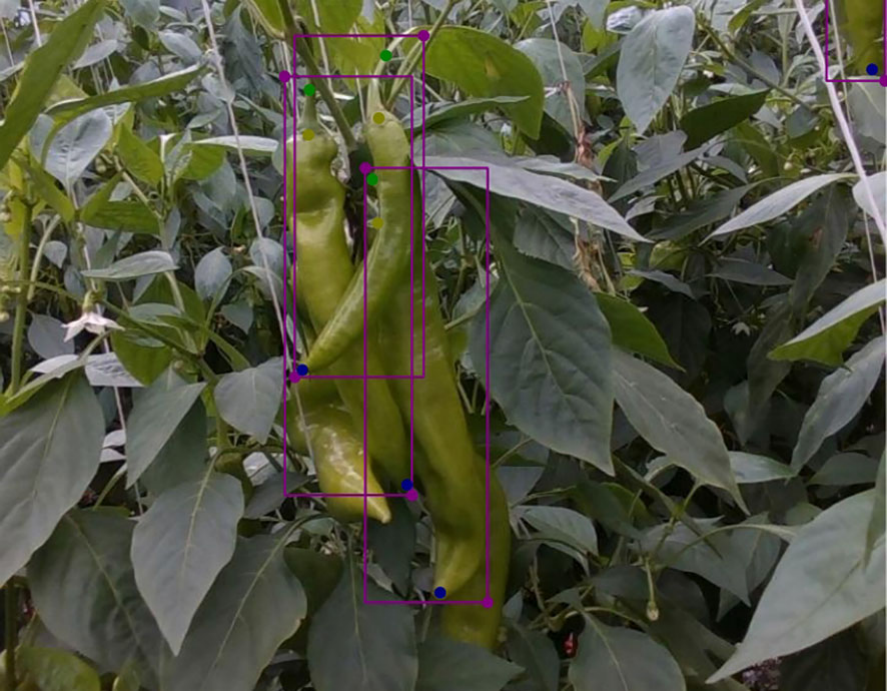
Overlapping phenomenon exists in the annotation boxes of peppers in complex scenes.

We observed that the accuracy of keypoints localization has consistently been lower than that of category detection, with some keypoints showing significant errors, as shown in **Figure 7**. In complex scenes, multiple peppers within the camera’s field of view often overlap, causing significant overlap in the bounding boxes as well, as illustrated in **Figure 8**. Before calculating the error using Equations 8, 9, the intersection-over-union (IoU) metric was used to match predicted boxes with ground truth boxes. However, due to the overlap of bounding boxes, this process sometimes resulted in incorrect matches between predicted and ground truth boxes. This erroneous matching increased the keypoint localization errors. Since the purpose of this study is to enable precise harvesting, where peppers are picked one by one, we propose a prioritized annotation strategy to address this issue. The strategy involves annotating only the peppers in the front when there is overlap between instances. Additionally, for instances where the pixel error exceeds 100, a second round of detection or a change in camera angle could help re-predict keypoint locations more accurately.

Notably, Pepper-YOLO achieves a lightweight design while maintaining high accuracy. Compared to YOLOv5n-Pose, its parameter count is reduced by 64.8%, and its model complexity is lowered by 19.2%. When compared to YOLOv8n-Pose, Pepper-YOLO reduces the parameter count by 38.3% and model complexity by 28.9%, all while improving detection accuracy. This low-resource model is particularly important for use in low-cost devices such as agricultural robots, where it can meet real-time detection demands and achieve precise operations in hardware-constrained environments. The reduced resource consumption makes it an ideal solution for enabling efficient and accurate real-time performance on low-cost agricultural robots and similar devices.

## 5 Conclusions

In this study, we successfully developed an improved lightweight Pepper-YOLO model, specifically designed for simultaneously detecting green peppers and their picking points in complex agricultural environments. First, we restructured the backbone network using a reversible multi-layer feature fusion module, allowing Pepper-YOLO to ensure lossless information transfer between different feature layers, significantly enhancing the model’s ability to handle occlusions and complex backgrounds. Second, by integrating RepNCSPELAN4 into the feature fusion process, the model’s multi-scale feature representation capabilities were greatly enhanced, enabling more accurate detection of peppers at different scales. Lastly, replacing the CIB module with the C2fCIB module optimized the recognition of large pepper features. Experimental results show that Pepper-YOLO achieved 81.1% accuracy in detecting green peppers and 88.1% accuracy in identifying picking points, with low Euclidean distance errors for picking point localization. Importantly, the model achieved this performance while reducing parameters by 38.3% and decreasing model complexity by 28.9%, resulting in a compact 4.3MB model, making it highly suitable for deployment on low-cost agricultural robots.

While the improved model is capable of real-time detection of green peppers and picking point localization in complex environments, its generalization to different pepper varieties and varying lighting conditions needs further validation. Future work will focus on expanding the data range to include more pepper varieties and diverse lighting conditions.

## Data Availability

The raw data supporting the conclusions of this article will be made available by the authors, without undue reservation.
